# Pilot of a person-centred, interdisciplinary approach to goal setting in Ireland’s National Rehabilitation Hospital: a study protocol

**DOI:** 10.12688/hrbopenres.13700.2

**Published:** 2026-07-21

**Authors:** Lauren Christophers, Zsofia Torok, Catherine Cornall, Aine Carroll

**Affiliations:** 1 School of Medicine, University College Dublin, Dublin, Ireland; 2 National Rehabilitation Hospital, Dublin, Ireland

**Keywords:** goal setting, person-centered care, interdisciplinary teams, rehabilitation, shared decision-making, interprofessional collaboration

## Abstract

**Background:**

Research has emphasized the value of a person-centered, interdisciplinary team (IDT) approach to structured goal setting in rehabilitation; yet these approaches are not consistently implemented in clinical practice, limiting the effect that goal setting can have on rehabilitation. The introduction of a new IDT, person-centered goal setting process to Ireland’s national rehabilitation hospital offers an opportunity to gain novel insight into barriers to implementing and normalising IDT goal setting in complex, specialist rehabilitation services. This process was developed prior to, and independently of, the current evaluation by the hospital’s quality improvement team; this protocol concerns the evaluation of its pilot implementation, not the development of the process itself.

**Methods:**

The goal setting process was collaboratively designed by the National Rehabilitation Hospital’s IDT Quality Care Team, prior to and independently of this study, based on iterative rounds of patient and staff feedback. It will be piloted in three different units of the hospital. The outlined study was co-designed by embedded academic researchers and knowledge stakeholders (the quality improvement team responsible for introducing the new process; and two former patients). The aim is to use a mixed methods approach to capture patient and staff experiences and perspectives of the new process and secondarily to pilot the feasibility of quantitative outcome measures ahead of a possible future hospital-wide evaluation. Data will be collected by embedded researchers with a combination of self-report measures and qualitative interviews with both staff and patients. Self-report measures focus on capturing person centeredness of goals, shared decision making, interprofessional socialization and process normalization. Quantitative data will be represented using descriptive and inferential statistics; qualitative data will be analysed using reflexive thematic analysis.

**Discussion & Conclusion:**

From this data the researchers aim to present a holistic view of the patient and staff experiences of the goal setting process. This may further illuminate challenges and potential solutions to implementing changes to goal setting within this context. Findings will be used to inform implementation within the hospital and will contribute to knowledge for rehabilitation practice around implementing IDT goal setting. This will add an Irish perspective to existing goal setting rehabilitation literature.

## Introduction

Rehabilitation is delivered by multi professional expert teams, focused on delivering interventions designed to optimize functioning and reduce disability in individuals with health conditions in interaction with their environment ( WHO, 2023). With these holistic and multidimensional aims, rehabilitation care is delivered by many different professionals who work together with varying levels of integration and collaboration. Where a multidisciplinary team (MDT) involves different disciplines working together with team members responsibilities focused on their own disciplinary remit, each discipline typically setting and pursuing its own goals with limited shared planning, an interdisciplinary team (IDT) involves more blurring of disciplinary boundaries ( Ellis & Sevdalis, 2019; Karol, 2014). An IDT includes members coming together, developing joint service plans and integrating closer to complete shared goals ( Ellis & Sevdalis, 2019). In the context of rehabilitation, goals are purposefully created and refer to a desired future state to be achieved by a person with a disability using rehabilitation activities and interventions ( Levack *et al.*, 2015).

Thus, an IDT approach to goal setting is focused on creating shared goals across disciplines and has been advocated for as multiple goals set by different disciplines with patients can create confusion and miscommunication among patients and team members. Evidence based consensus statements recommend an IDT approach including goal setting for rehabilitation following traumatic brain injury for better outcomes ( Grabljevec *et al.*, 2018; Miller *et al.*, 2010). A critical review comparing factors impacting functional outcome for stroke found interdisciplinary models enhanced outcomes comparative to MDT models ( Cifu & Stewart, 1999). However, the vast majority of goal setting in rehabilitation care is multidisciplinary in nature as demonstrated in surveys, reviews and ethnographic observations across the UK and Australian rehabilitation services ( Baker *et al.*, 2022a; Scobbie *et al.*, 2015). How exactly an IDT can best work together to create common goals is unknown.

A shift from multidisciplinary teams to interdisciplinary teams also represents a shift from being discipline-oriented to being person focused ( Karol, 2014). For goal setting this is another important factor, the degree of “person centeredness” of the goals. Person centered care is focused on meaningful outcomes for an individual based on their life and their preferences ( Håkansson Eklund *et al.*, 2019) and is underpinned by values of mutual respect, individual right to determination and compassion ( McCormack *et al.*, 2010; McCormack & McCance, 2016). Although a person-centered approach to goal setting takes time and effort, it has been identified as worthwhile as centering practice around a person’s goal can provide clear structure for rehabilitation efforts, increase patient motivation and IDT communication, enhance patient engagement and is associated with goal attainment ( Doig *et al.*, 2009; Ownsworth *et al.*, 2008; Turner-Stokes *et al.*, 2015). A person-centered approach that involves the patient in decisions about their goals is a process of shared decision making between the patient and the team members. Patients often report preferring a shared decision-making approach, though they can feel ill equipped or under prepared to do so, particularly at the start of the inpatient rehabilitation journey ( Baker *et al.*, 2022a; D’Cruz *et al., *2016; Rose *et al.*, 2017; Rose *et al.*, 2019). It is important that patients are equipped with relevant information both early on and throughout the goal setting process, which often does not occur ( Scobbie *et al.*, 2015).

Thus, a best practice approach to goal setting is one that involves shared decision making with the patient, creating person centered goals to be shared by the rest of the team. Though there is evidence to indicate that patients who are more involved in the goal setting process experience greater improvements in their function ( Dalton *et al.*, 2012; Turner-Stokes *et al.*, 2015), shared decision making with the person during goal setting has been limited in clinical practice. Clinicians do not consistently use shared decision making in goal setting ( Cameron *et al.*, 2018; Rose *et al.*, 2017). Another reported patient preference is a structured approach to goal setting. For example, the commonly cited Goal setting and Action Planning (G-AP) framework incorporates useful strategies for enhancing goal setting such as action and coping planning, and goal review and feedback ( Scobbie *et al.*, 2011). These strategies are also inconsistently implemented in practice, with a common absence of review and feedback in inpatient rehabilitation ( Plant & Tyson, 2018) and only 60% of UK community rehabilitation services reporting breaking down goals to action plans ( Scobbie *et al.*, 2015).

There are clearly challenges to implementing and normalising best practices around goal setting. While the need for training and education for staff and patients has been highlighted ( Rose *et al.*, 2017), the challenges of implementing and normalizing a collaborative, person centered, shared decision-making goal setting approach in rehabilitation are manifold. It is clearly difficult to introduce major changes to teamwork where most rehabilitation professionals work in a multidisciplinary rather than interdisciplinary way, where shared decision making requires time and additional supports for the patient and when rehabilitation teams are also adhering to organizational constraints and guidelines. Implementing goal setting interventions is a complex endeavor. Healthcare systems are characterized by being complex and adaptive, with different interacting agents at different levels influencing any change ( Carroll, 2021; Peters, 2014). Thus, research must focus on gaining insight into factors that can enable organizations and individuals to successfully implement changes in goal setting processes in line with what the literature has so far identified as best practice.

To this end, some researchers have explored implementation of strategies to support goal setting and how best to introduce and maintain a collaborative, shared decision-making approach. Scobbie *et al.* (2020) examined implementation of the G-AP framework in three community rehabilitation teams identified barriers such as an organizational set up that did not facilitate the process, lack of leadership support and team engagement and negative staff appraisal of the impact of practice change. Van de Weyer *et al.* (2010) explored perspectives around a keyworker led goal setting practice involving increased patient involvement and shared decision making. They reported barriers such as time needed to facilitate new practice, staff work patterns and staff level of skill. Doig *et al.* (2023) conducted qualitative interviews with professionals who trialed a role based, interdisciplinary goal setting process in inpatient rehabilitation for individuals with traumatic brain injury. Staff reflections emphasized the importance of putting the person at the centre, accepting the mind shift to participation focused goals and working collaboratively. The central challenge was described as the challenge of unlearning discipline specific goal setting ( Doig *et al.*, 2023). Baker, Cornwell, Gustafsson, Stewart, *et al.* (2022b) used a co-design approach to implementing IDT goal setting and through focus groups identified barriers relating to clinicians lacking knowledge and understanding around goal setting terminology and processes. Following this, co-designed interventions for behavioral change included staff training modules, client held workbook and interdisciplinary goal conference templates and educational rehabilitation service fliers.

These studies have largely been conducted in the UK or Australia and have focused on specific diagnostic populations or on community rehabilitation. The generalizability of these findings to an Irish context is uncertain. It is likely that the implementation of complex interventions such as IDT goal setting requires tailoring to local context ( Baker *et al.*, 2022b; Craig *et al.*, 2013). Existing frameworks such as G-AP ( Scobbie *et al*., 2020), the keyworker-led model ( Van De Weyer *et al*., 2010), and role-based IDT goal setting ( Doig *et al*., 2023) were each developed for single-diagnosis or community rehabilitation populations. The NRH is Ireland’s only complex specialist rehabilitation hospital, serving a mixed diagnostic inpatient case-mix across acquired brain injury, stroke, spinal cord injury, limb absence, and paediatric programmes, within an existing structure that already includes formal family meetings. Rather than adopt a single-diagnosis framework unmodified, the hospital’s quality improvement team chose to adapt core best-practice principles identified in this literature; structured goal capture, action planning, regular review, and shared decision-making, into a process tailored to this mixed case-mix and to the hospital’s existing structures (see Intervention, below). The current study will add new perspectives to the literature by focusing on experiences of IDT goal setting from both staff and patients, in the context of an in-patient case-mix service in Ireland. From this, new insight may be gained into factors that can enable organizations and teams to successfully implement changes in goal setting and team working.

To summarise, best practices in goal setting in rehabilitation have been identified, but translating this into clinical practice has proven challenging. Given these obstacles, it is imperative that research continue to explore and identify solutions to the obstacles for successful implementation and normalization. In line with this, the outlined study will focus on the introduction of a tailored shared decision-making, interdisciplinary goal setting process in a mixed diagnostic case, inpatient rehabilitation setting – Ireland’s National Rehabilitation Hospital (NRH). To be explicit about the scope of this protocol: this study evaluates the pilot implementation of a goal-setting process that was co-designed and finalised prior to this evaluation; it is not a study of the development of that process, nor a naturalistic study of pre-existing, unstructured goal-setting practice. The constructs under evaluation, and how each is assessed, are summarised in Table 1 above.

**Table 1.** Constructs evaluated in this study and corresponding methods.

Construct	Definition used in this study	How it is evaluated
Interdisciplinary teamwork	Team members work jointly across disciplinary boundaries toward shared goals (see Introduction for MDT/IDT distinction)	Staff interviews (qualitative); ISVS-9B (interprofessional socialisation, quantitative)
Shared decision-making	Patient and team jointly determine goals and priorities	Patient interviews (qualitative); CollaboRATE (quantitative)
Person-centredness	Goals reflect the individual’s own priorities, values and life context	Patient interviews (qualitative); C-COGS (quantitative)
Goal setting	The structured process of identifying, documenting and reviewing rehabilitation goals	Staff and patient interviews; process documentation reviewed as part of the intervention description
Implementation/normalisation	The extent to which the new process becomes embedded in routine practice	Staff interviews (qualitative); NoMAD (quantitative)

### Research questions, aims and objectives

*Research Question 1: What is the patient experience and perspective of a shared decision making, interdisciplinary goal setting process in a rehabilitation hospital?*

*Research Question 2: What is the staff experience and perspective of a shared decision making, interdisciplinary goal setting process in a rehabilitation hospital?*

Guided by these research questions, we intend to capture the patient experience of the new process, explore the degree of person centeredness of patients’ goals and whether the patient feels included in decisions about their care. From a staff perspective we will qualitatively explore their experience of the new process as well as how the process relates to their interprofessional values and socialisation. The quantitative measures address these same research questions from a complementary angle: rather than seeking new research questions of their own, C-COGS and CollaboRATE provide a standardised, repeatable measure of person-centredness and shared decision-making for Research Question 1, and the ISVS-9B and NoMAD provide standardised, repeated measures of interprofessional socialisation and normalisation for Research Question 2. This allows patterns identified qualitatively to be considered alongside a numeric, comparable account of the same constructs (see Study design and Data analysis, below).

*Research Question 3: What are the barriers and enablers to implementing and normalising this process in a rehabilitation hospital?*

Guided by this research question, we will use the data collected to identify barriers and enablers to implementation of an IDT goal setting process. These barriers and enablers can later be used to co-design behavioural interventions and further supports to facilitate hospital wide implementation of the goal setting process. This will likely be the subject of a later study by the project team.

Data will be collected by two embedded researchers. Embedded researchers have been defined as knowledgeable researchers working with a team that will be responsible for implementing changes ( Churruca *et al.*, 2019). During the piloting of a shared decision-making IDT goal setting process within Ireland’s NRH, embedded researchers (LC and ZT) will a) capture staff and patient experiences of this new process, and b) identify potential barriers and enablers to implementation within the hospital, including responsibility for recruitment and eligibility screening of participants (see Eligibility criteria, below). Learning from this will not only be disseminated into the literature base, but feedback will be utilised by the quality improvement team within the hospital who are responsible for finalising and implementing a new goal setting procedure (see “Public and patient involvement”).

## Methods

### Study design

This study will use a mixed methods approach and is focused on the piloting of a shared decision making, interdisciplinary goal setting process with three teams in Ireland’s National Rehabilitation Hospital. By capturing staff and patient experiences using multiple and mixed methods, we can gain insight into the phenomenon of shared decision making, IDT goal setting from different perspectives as well as barriers to implementation in this context. Qualitative and quantitative data will be collected from consenting staff and patients involved in the pilot, using semi structured interviews and self-report measures. The two strands serve complementary but distinct purposes. The qualitative interviews are designed to answer Research Questions 1–3 directly, eliciting patient and staff experience of the process in depth. The quantitative self-report measures serve two secondary purposes: first, to provide a standardised, repeatable measure of staff interprofessional socialisation (ISVS-9B) and process normalisation (NoMAD), and of patient-perceived client-centredness (C-COGS) and shared decision-making (CollaboRATE), which can be compared descriptively across time points and considered alongside the qualitative account; and second, to pilot the feasibility and acceptability of these instruments, developed and validated in Australian and North American settings, for possible use in a future, hospital-wide evaluation of the goal setting process. Given the small anticipated sample, the quantitative strand is treated as descriptive and exploratory rather than as a primary hypothesis test. This is a convergent mixed-methods design: quantitative and qualitative data will be analysed separately and then integrated at the interpretation stage (see Data analysis). It should be noted that elements of this study design may be subject to change due to organisational constraints and factors beyond the researcher’s control. Any changes to the study design will be registered with the protocol.

### Study setting

This study will take place in the National Rehabilitation Hospital (NRH), Ireland, a 120 bedded rehabilitation hospital. The NRH is a national, specialist, tertiary university hospital providing complex specialist rehabilitation services to adults and children who have an acquired illness or injury. The NRH is a part of the Ireland East Hospital Group and Ireland’s Integrated Rehabilitation pathway with close clinical links with national spinal and neurosurgical centres at acute hospitals. As Ireland’s only complex specialist rehabilitation hospital the NRH accepts patients from hospitals and health care facilities throughout the country. Consultant led interdisciplinary teams provide speciality programmes in acquired brain injury; stroke; spinal cord system of care (SCSC); prosthetic, orthotic and limb absence rehabilitation (POLAR), and paediatric family-centered rehabilitation. The Stroke, POLAR and paediatric programmes each have one in-patient unit, SCSC programme has three units, and the Brain Injury programme has four units. The hospital is transitioning from paper healthcare records to an electronic patient record system (EPR). The new goal process designed for an EPR is being introduced initially in paper format.

### Eligibility criteria

The study will recruit participants among the patients and staff of the National Rehabilitation Hospital. A convenience sampling approach will be used: all eligible staff on the three participating units, and all eligible patients admitted to those units during the recruitment window, will be invited to participate. Recruitment and determination of eligibility will be carried out by the two embedded researchers (LC and ZT), in liaison with each unit’s clinical lead.

Eligible patient participants will be the inpatients receiving rehabilitation in the NRH, admitted to one of the units enrolled in the intervention study, after the introduction of the new goal setting process. Where a patient has a cognitive or communication impairment, the treating team will first consider whether existing, hospital-approved communication supports (for example communication passports, augmentative and alternative communication aids already in use for that patient, or a family/carer-supported interview) can enable safe and meaningful participation. Patients will be excluded only where these supports are judged clinically insufficient to gain valid consent and to elicit the patient’s own view.. Staff members eligible for study participation are members of the interdisciplinary team on a participating unit directly involved in the goal setting process, including medical, nursing, and health and social care professional (HSCP) staff (for example physiotherapy, occupational therapy, speech and language therapy, social work and psychology) and healthcare assistants, working on that unit during the time of the trial. Administrative and non-clinical staff are not included.

### Intervention

This study will focus on the pilot of a new goal setting process in Ireland’s National Rehabilitation Hospital. This process was developed prior to, and independently of, this evaluation study, by the National Rehabilitation Hospital’s IDT Quality Care Team. The new goal process was developed in response to feedback from and in collaboration with NRH staff and patient representatives. Development took place over a two-month co-design period involving weekly workshops with a wider co-design group comprising three academic researchers (including the two embedded researchers responsible for this evaluation, LC and ZT), two former patients, and staff representatives from medicine, nursing and HSCP disciplines (see Public and Patient Involvement). In the implementation of complex healthcare interventions, it is likely that a co-design approach with tailoring to specific contextual barriers and enablers will be more successful ( Craig *et al.*, 2013). The new goal process was created with this in mind, informed by iterative rounds of feedback from staff and patients. As set out in the Introduction, existing goal-setting frameworks reported in the literature (e.g., Scobbie *et al*., 2020; Van De Weyer *et al*., 2010; Doig *et al*., 2023) were developed for single-diagnosis or community rehabilitation populations; the co-design group instead adapted core best-practice principles from this literature into a process tailored to the NRH’s mixed diagnostic case-mix and its existing structures, including its established family meeting model. It spans the rehabilitation journey from pre-admission to the NRH inpatient rehabilitation programmes to discharge and life after the NRH. Documentation to capture goals and the interdisciplinary care plan was changed to meet the requirements of the introduction of the EPR.

The change of the goal setting process is being accomplished in an iterative manner, based on the principles of person-centredness, partnership and patient goals driving the rehabilitation activities and schedule. Prior to admission, the goal process will be introduced to the patient and/or family through contact with members of NRH staff providing both written (a single information booklet used across all specialties, with a short specialty-specific insert) and verbal information. Gathering the person and family’s story—in order to getting to know the person and what matters most to them—will capture the patient’s or their representative’s understanding of their health condition, hopes and visions for life after rehabilitation and their three to five identified goals for their admission, including their perception of the importance of each goal and their proximity to achieving it. Where a patient is unable or does not wish to lead their own goal-setting, a named key worker (typically from nursing or allied health) leads goal capture collaboratively with the patient and family, adjusting the level of support to the patient’s stated preference and capacity. Recognised tools and accessible supports used at the ‘meet the team’ session include the hospital’s existing communication aids, for example Talking Mats, easy-read materials, and interpreter services where required.

The initial goals will be discussed during a goal capturing meeting held within the first two–five days of admission. As soon as practical within the first two weeks, the goal, action and care planning meeting meeting (the goal-setting conference) will take place, where the patient and/or family will meet with key members of the interdisciplinary team (medical, nursing; HCA and relevant HSCP staff ) to formally document the patient’s long-term goals in the EPR. The team, together with the patient and/or family then collaborate on identifying the short-term goals that underpin each longer-term goal identified. The new documentation captures the patients’ identified goals; the short-term goals underpinning the main goals; tasks and actions to be completed by both IDT members and the patient and factors that will support or interfere with achievement. Establishing peer patient group sessions to discuss long and short-term goals will be introduced and will take place on a regular basis.

**Figure 1.**
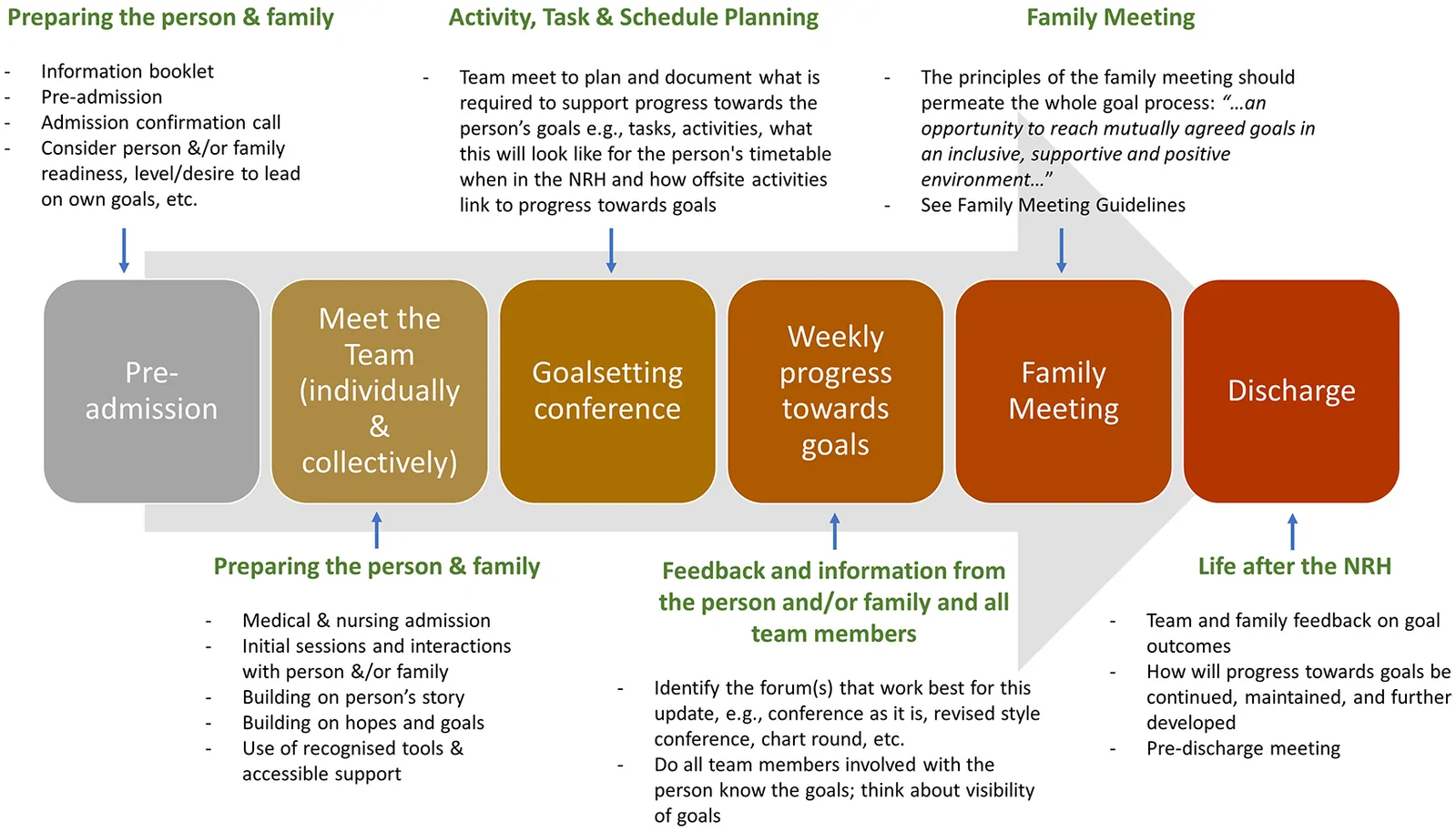
The new IDT goal setting process.

Reviewing and revisiting goals will be a continuous process that is both formal and informal and includes the person and/or their family. New short-term goals can be added at any time and the patient may also revise their long-term goals during any of their goal review meetings and discussion. The interdisciplinary team review on a weekly basis may also include longer sessions with the patient and family, incorporating the patient’s and/or family’s own feedback on progress towards goal outcomes and can be complemented by intermittent formal IDT review meetings with the patient and/or family ( Figure 1).

Staff training accompanied the roll-out of the new process on each unit. This comprised system-wide briefing sessions delivered by the IDT Quality Care Team prior to go-live on each unit, covering the rationale for the new process, the revised documentation, and worked examples of person-centred goal capture. Unit-based staff champions were identified on each participating unit to provide ongoing peer support during the pilot period.

A structured description of the intervention, mapped against the TIDieR checklist ( Hoffmann *et al*., 2014), is provided in Table 2.

**Table 2.** TIDieR description of the goal-setting process intervention.

Item	Description
Brief name	New interdisciplinary, person-centred goal setting process
Why	To replace discipline-siloed goal setting with a shared, person-centred, interdisciplinary process spanning admission to discharge (see Introduction)
What (materials)	Pre-admission information booklet (with specialty-specific insert); revised EPR/paper documentation for long- and short-term goals, tasks and actions; communication aids (e.g., Talking Mats, easy-read materials)
What (procedures)	Pre-admission contact; ‘meet the team’ session; goal capturing meeting (days 2–5); goal, action and care planning meeting/goal-setting conference (within 2 weeks); weekly IDT review; family meetings; peer patient group sessions; pre-discharge meeting
Who provided	Interdisciplinary team members (medical, nursing, HCA and relevant HSCP staff ), with a named key worker where a patient requires additional support to lead their own goal-setting
How	Face-to-face, individually and collectively (team conferences, family meetings)
Where	Three inpatient units of the National Rehabilitation Hospital
When and how much	Spans pre-admission to discharge and follow-up; goal-setting conference within 2 weeks of admission; weekly review thereafter
Tailoring	Adapted from existing goal-setting frameworks in the literature to the NRH’s mixed diagnostic case-mix and existing family meeting structure (see Introduction, Intervention)
Modifications	Any changes made during the pilot will be logged and reported in the results paper

### Data collection

***Quantitative data***

*Measures*

For patients the self-report measures (C-Cogs and CollaboRATE) will be administered shortly after the goal, action and care planning meeting (the goal-setting conference described above, occurring within the first two weeks of admission; distinct from the earlier ‘meet the team’ session) (see intervention description). These measures are designed for use after a specific consultation and are therefore best used in this way. For patients who wish to complete these measures but face literacy, fatigue or motor barriers to doing so unaided, researchers (LC/ZT) are available to read items aloud, clarify wording, or transcribe responses. For staff participants the ISVS-9B will be administered at three time points (prior to the trial, after new goal setting process has begun, and at the conclusion of the trial) and the NoMAD administered alongside it at time three. See Table 3 below for data collection time points.

**Table 3.** Data collection timeline.

	Time 1: before trial	Time 2: after initial goal setting meeting	Time 3: after trial
Staff measures	ISVS-9B	ISVS-9B	Semi structured interview; ISVS-9B; NoMAD
Patient measures	N/A	C-Cogs and CollabORATE	Semi structured interview

*Patient measures*

C-Cogs

The Client-Centredness of Goal Setting (C-COGS) ( Doig *et al.*, 2015) is a 13 item instrument designed to measure the degree of client-centredness of goal-planning approaches from the client’s perspective. It has three subscales (Alignment, Participation and Goals) and is scored on a 5-point Likert scale (1 = Strongly disagree, 5 = Strongly agree). The participation sub-scale measures the person’s perceived participation in the goal setting process and can be scored separately (range 6–30). The goals sub-scale measures the ownership, importance, meaningfulness and relevance of the individual rehabilitation goals, and the average is calculated across the goals (range 4–20). A total score can be generated by adding the sub-scale scores, higher total C-COGS scores indicating higher levels of client-centredness of goal planning. The C-COGS is characterised by good internal consistence (Cronbach alpha = 0.82) ( Prescott *et al.*, 2019).

CollaboRATE

The CollaboRATE ( Barr *et al.*, 2014) is a short patient-reported measure of shared decision making that patients complete following the goal planning meeting. It contains three brief questions, measuring the dimensions of the health issue, the elicitation, and the integration of patient preferences. It is rated on 5-point Likert scale (0 = No effort was made, 4 = Every effort was made), scores ranging from 0–12. Higher scores indicate greater efforts made to support the patient’s participation in the shared decision-making process. The CollaboRATE evidences concurrent validity with other measures of shared decision-making and excellent intra-rater reliability ( Barr *et al.*, 2014).

*Staff measures*

ISVS-9B

The ISVS-9B is the short version of the original 21 item Interprofessional Socialization and Valuing Scale (ISVS-21) ( King *et al.*, 2016). It is a self-report instrument, assessing individuals’ beliefs, behaviours, and attitudes and is designed to measure interprofessional socialization and readiness to function in interprofessional teams. The three subscales reflect three key concepts of interprofessional practice: roles (belief ), client-centeredness (attitudinal items), and conflict/negotiation (behavioural items). The scale was developed to evaluate the effect of interprofessional education and other interventions, in a pre-post-test study design. It is rated on 7-point Likert scale (7 = To a Very Great Extent, 1 = Not at All) and contains the option of choosing the “N/A” (not applicable). The scores are ranging from 0–63, higher scores indicating more positive views and higher expression of interprofessional socialization behaviours. The ISVS-21 is characterised with an excellent internal consistency (Cronbach alpha = 0.98).

NoMAD

The Normalization Measurement Development questionnaire (NoMAD) ( Finch *et al.*, 2018) is a 23-item instrument, measuring the normalisation of complex healthcare interventions. It is based on normalisation process theory (NPT) and was developed for evaluating the status or outcome of an implementation process and the sustainability of the intervention. The first part contains three general questions about the intervention, the second consists of 20 items reflecting the four NPT constructs (coherence, cognitive participation, collective action and reflexive monitoring). The questions in the second part have two categories of answers: Option A has five options (strongly disagree–strongly agree), while the Option B participants can choose if the statement is not relevant. While the NoMAD does not have a defined scoring guide, it is often scored on a five-point Likert scale (1 = strongly disagree, 5 = strongly agree) ( Gillespie *et al.*, 2018; Lamarche *et al.*, 2022). The NoMAD is characterised with good psychometric properties, demonstrating sufficiently high levels of internal consistency along the four dimensions (Cronbach alpha = 0.89).

***Qualitative data***

Qualitative data will be collected from participating patients at the conclusion of the pilot. Semi-structured interviews will be conducted with participants autonomy and control over the discussion reflected. A topic guide has been prepared for the interviewer, but this will be flexible to each participant, and largely consists of open-ended questions designed to encourage participants to share experiences from throughout their goal setting process within the hospital. The topic guide was prepared by the embedded researchers, presented to the co-design team and specifically reviewed by two ex-patients on the team.

Qualitative data will be generated from staff also using semi structured interviews. Reflective questions will be asked about interdisciplinary teamwork and interprofessional collaboration, person centered care and shared decision making and the goal setting process itself. These key topics will be explored with open ended questions. Questions for staff have been drafted by researchers, presented to the co-design team and specifically reviewed by staff members from different disciplines.

### Data management

All collected data will be safely stored and used in accordance with the Data Protection (Amendment) Act of 2003 and General Data Protection Regulation (GDPR) 2016/679. Participants’ data will be de-identified and will be presented in in reports and publications either in aggregate form or, where necessary, using pseudonyms.

### Data analysis

***Quantitative data***

Data from self-report measures will be imported into SPSS (IBM SPSS Version 27; an open access alternative is R, or R Studio). Descriptive statistics will be used to illuminate the means, standard deviations etc, of the patient measures – C-Cogs and its subscales as well the 3-item collaboRATE. Given the small sample of patients undergoing the trial, more comprehensive or inferential statistics are not appropriate (Maximum potential sample size of 16 patients). However, these measures can be used complementary to qualitative data to gain insight into the patient experience of the new goal setting process. Furthermore, the study offers the opportunity to pilot these measures with a view to potentially using them in a hospital wide impact study in the future.

For the staff self-report measures, inferential statistics can be used to test the following hypothesis:

H1a: Interprofessional socialisation and values scores will significantly change over the course of the trial.

A one-way repeated measures analysis of variance (or suitable non-parametric alternative) can be used to test whether scores significantly change over time. An a priori power analysis was conducted using G Power and indicates that with an alpha level of 0.05, a sample of 27 staff members would be required to achieve 80% power in detecting a medium effect size. Descriptive statistics will be calculated for the staff NoMAD data – in order to investigate the perspective of staff on normalisation and implementation of this new goal setting process across the hospital.

Mixed-methods integration

This is a convergent mixed-methods design. Quantitative and qualitative data will be analysed separately using the methods described above, and integrated at the interpretation stage through a narrative weaving approach, using the qualitative findings to help explain and contextualise the quantitative results (for example, using interview data to interpret patterns observed in NoMAD subscale scores), rather than a fully joint-display integrated analysis, which was judged disproportionate given the modest quantitative sample size.

***Qualitative data***

*Theoretical grounding*

The researchers will take a constructionist perspective, with a critical orientation to the analysis of qualitative data. Based on this we have selected reflexive thematic analysis as the primary method of analysis. We recognise language as an implicit tool in socially producing meaning and understanding of experiences. In this view, participants in interviews are not merely sharing their experiences but rather reflecting upon memories and constructing meaning as topics are discussed. A critical orientation to analysis means that we understand firstly, the artificial and constructed nature of the interview but also that we aim to see beyond language. If language creates one’s subjective reality, then we aim to offer interpretations of meaning beyond those explicitly stated by the participants. In the language of reflexive thematic analysis – this means including latent codes alongside semantic ones, taking an inductive approach in conjunction to a deductive one and keeping a critical perspective rather than a more essentialist one.

*Analysis method*

Based on the theoretical grounding outlined above and the research questions, the co-design team have selected reflexive thematic analysis as the most appropriate method of qualitative data analysis. The lead researcher (LC) will cycle through Braun and Clarke’s six phases in an iterative, exploratory approach (familiarisation with data, generating initial codes, generating themes, reviewing themes, defining and naming themes, reporting themes) ( Braun & Clarke, 2006, Braun & Clarke, 2021; Terry *et al.*, 2017). Following transcription, the researcher will read and re-read transcripts while listening to audio recording, thus immersing herself in the data. The lead researcher (LC) will import the data to NVivo and will begin initial coding. A predominantly inductive approach will be taken with open coding on both patient and staff interview data. Codes will include semantic codes which summarise a data segment and latent codes which interrogate a deeper, underlying meaning behind a data segment. This will be based on the interpretation of the researcher. With the generation of a set of initial codes, similar meaning units will be clustered to create themes and sub-themes. From here we aim to use this analysis to construct an understanding of the patient and staff experience of the trial. For staff, this will include attitudes and expectations prior to the trial as well as reflections after the trial through using two interviews. For patients, the goal setting journey is elicited through reflections during one interview at the conclusion of the trial.

### Ethics

Ethical approval for this study has been granted by the NRH Research Ethics Committee (date of approval 24/05/2022) and the UCD Office of Research Ethics (Research Ethics Exemption Reference Number LS-E-22-06-Carroll, date of approval 13/01/22).

### Public and patient involvement

This study was co-designed by embedded academic researchers and knowledge stakeholders. The co-design team includes two former patients, members of the NRH’s IDT Quality Care Team who are responsible for organizing the implementation of new goal setting processes, and three academic researchers. The new goal setting process itself was designed by this co-design group prior to, and independently of, this evaluation study; the same group also collaboratively decided on the research questions, measures and methods for the evaluation described in this protocol. This study was rapidly co-designed over a two-month period. The co-design team met weekly and collaboratively decided on appropriate research questions, measures and methods outlined in this document.

The involvement of clinicians and former patients goes beyond stakeholder consultation, as the co-researchers are contributing not only clinical expertise and knowledge alone butactively co-designed the research study collaboratively making decisions on measure selection, interview questions *etc..* There is evidence that involving patients not only brings lived-experience perspective into research but increases its quality and the relevance ( Brett *et al.*, 2014; Nierse *et al.*, 2012). Co-designing with healthcare professionals has been identified as a key factor in successful implementation of healthcare interventions and uptake of research findings ( Jessup *et al.*, 2018). The co-design team will continue to be involved throughout the research process and thus encourage organisational uptake of research findings.

### Dissemination of results

A bespoke dissemination plan will be developed. We will share the iterative process during the active phase of data gathering with the internal academic and clinical community. Upon completion we will share findings in public venues (library, hospital and university space), round table discussions (academic and clinical venues), and academic presentations and workshops both within and outside of the hospital community. The end users and stakeholders are the patient and carers, academic, clinical, and managerial staff who can take findings and initiate action to address the issues identified in the project. Academic and clinical educators can also utilize the project presentation to educate clinicians, students and goal setting. Results of this study will also be disseminated through publication in in peer-reviewed academic journals and on social media. Findings will be also presented at national and international conferences and to relevant stakeholder groups.

## Study status

This study is in progress, in line with introduction of the new goal setting process within the hospital. The baseline (time 1) data collection for staff is currently underway.

## Conclusions/Discussion

The value of a person-centered approach to goal setting in rehabilitation is clear – it can result in better functional outcomes and increased patient satisfaction. However, there have been issues in implementing new goal setting approaches to complex healthcare systems. Without investigating implementation of complex healthcare interventions, we run the risk of “shared decision making” or “interdisciplinary teams” or “person centered” becoming tokenistic phrases rather than real life practices. Indeed, research has identified that many rehabilitation teams describe themselves as interdisciplinary despite not meeting evidence-based criteria to qualify as one ( Baker *et al.*, 2022a), shared decision making is something that clinicians can describe but do not always use these skills in practice ( Cameron *et al.*, 2018); clinicians and teams do not consistently implement goal setting strategies into practice, limiting the effect that goal setting can have on rehabilitation ( Marsland & Bowman, 2010; Plant & Tyson, 2018; Scobbie *et al.*, 2015). The introduction of an interdisciplinary, person-centered goal setting process to Ireland’s national rehabilitation hospital offers an opportunity to gain novel insight into mixed case mix diagnosis rehabilitation services and the potential barriers to implementing and normalising such complex changes. Patient and staff experience and perspectives may further illuminate challenges and potential solutions to implementing changes to goal setting within this context. From this we hope to offer recommendations for rehabilitation practice around implementing changes to goal setting processes.

## Limitations

One limitation is the data collection time frame, which is set by organisational factors. The goal setting process and new documentation is due to be finalised in tandem with the introduction of an electronic patient record system within the hospital. This places constraints on the time that can be spent on data collection, though the co-researchers and authors have worked together to ensure that activities are feasible. A second limitation is the study sample, which may not be a wholly representative sample of staff. There is a possibility of recruitment bias whereby only people interested in changes in goal setting will participate. We also acknowledge limited participation of staff working night shifts or less flexible time schedules. Despite efforts to support participation through existing communication aids (see Eligibility criteria), patients with the most severe cognitive or communication impairments will still be unable to participate, and our patient sample will therefore under-represent this group. A small sample of patients is expected as a small number of admissions are anticipated in the time available for recruitment. However, the sample will be appropriate to achieve qualitative data saturation ( Hennink & Kaiser, 2022) from interview data. Finally, a potential limitation of this study is the questionnaires used with staff and patients which were developed in Australia and USA, and therefore have not been validated or adapted for use within an Irish context. A further limitation is that the co-design group that developed both the goal setting process and this evaluation protocol included two former patients but no family carers, and both patients had experience of a single diagnostic pathway rather than the full range of conditions seen at the NRH (including acquired brain injury, stroke, spinal cord injury, limb absence and paediatric rehabilitation). This may mean that both the process and this study design reflect that experience more than others; future iterations of this work should aim to widen patient and carer representation across the hospital’s full case-mix.

## Data availability

No data are associated with this article.
