# Supplementary material for: Pilot of a person-centred, interdisciplinary approach to goal setting in Ireland’s National Rehabilitation Hospital: a study protocol
Source: HRB Open Res. 2026 Aug 27;6:28. Originally published 2023 May 3. [Version 3] doi: 10.12688/hrbopenres.13700.3 (PMC13562921; doi:10.12688/hrbopenres.13700.3)
Supplement: Peer review history

# Reviewer report: Reviewer response for version 3

**DOI:** 10.21956/hrbopenres.16025.r57562

**Published:** 2026-09-12

**Authors:** António Lista

**Affiliations:** 1 Universidade de Évora, Évora, Portugal

Dear authors,

 This protocol addresses a relevant question in rehabilitation: how to involve people in setting their own goals and organise teamwork around those goals. The research questions are clear, and the combination of interviews and questionnaires seems appropriate. The changes made in response to previous reviews have helped clarify the intervention and the procedures for participation in the study.

 During my reading, a few questions remained that I would welcome the authors’ clarification on:

1. In Table 3, staff interviews appear only at the end of the pilot. However, the qualitative analysis section refers to two interviews, before and after the pilot. Are one or two interviews planned? If two, will the same staff members be interviewed at both time points? It would also be useful to specify the recruitment duration and the intervals between assessments. For patients, does the interview take place at the end of their admission or at the end of the overall pilot?
2. The quantitative component is described as exploratory, but a hypothesis and a sample size calculation are then presented to test changes over time. I do not see these choices as necessarily incompatible, but it would help to explain how they fit together and how the findings will be interpreted. Please also clarify which score will be analysed and how incomplete assessments and loss to follow-up will be handled. The description of NoMAD as a repeated measure should be revised, as it is scheduled for administration only at the end of the pilot.
3. The authors state that they intend to assess the feasibility and acceptability of the questionnaires for a future study. How will this be done? Will they collect participant feedback, record difficulties with completion, or examine missing responses? A brief explanation would help readers understand how this objective will be addressed.
4. The description of the goal-setting process is helpful, and the authors explain why it needed to be adapted to the hospital context. However, I would welcome a clearer explanation of how the cited frameworks informed the choices made. Which principles were used to develop the process, and how are these reflected in the person’s participation, the negotiation of goals, and the review of the plan?

Finally, the Study Status section seems to refer to an earlier stage than that described in the responses to reviewers. 

 I suggest updating this statement. It would also be helpful to include a direct link to the interview guides mentioned as supplementary material.

 I believe this study could make a useful contribution to rehabilitation practice. The clarifications above would help readers understand what is planned and resolve some inconsistencies between sections of the protocol.

Is the study design appropriate for the research question?

Yes

Is the rationale for, and objectives of, the study clearly described?

Yes

Are sufficient details of the methods provided to allow replication by others?

Partly

Are the datasets clearly presented in a useable and accessible format?

Not applicable

Reviewer Expertise:

Rehabilitation nursing; ageing; functional assessment; person-centred care; quantitative research methods and evidence synthesis.

I confirm that I have read this submission and believe that I have an appropriate level of expertise to confirm that it is of an acceptable scientific standard, however I have significant reservations, as outlined above.

# Reviewer report: Reviewer response for version 3

**DOI:** 10.21956/hrbopenres.16025.r57543

**Published:** 2026-09-03

**Authors:** Yvonne Codd

**Affiliations:** 1 Trinity College Dublin, Dublin, Ireland

No further comments to make.

Is the study design appropriate for the research question?

Yes

Is the rationale for, and objectives of, the study clearly described?

Yes

Are sufficient details of the methods provided to allow replication by others?

Partly

Are the datasets clearly presented in a useable and accessible format?

Partly

Reviewer Expertise:

My background and research experience are in enduring health conditions, service design, and rehabilitation research, including the design and evaluation of studies examining person-centred rehabilitation outcomes and clinical practice.

I confirm that I have read this submission and believe that I have an appropriate level of expertise to confirm that it is of an acceptable scientific standard.

# Reviewer report: Reviewer response for version 2

**DOI:** 10.21956/hrbopenres.15995.r57016

**Published:** 2026-08-10

**Authors:** Yvonne Codd

**Affiliations:** 1 Trinity College Dublin, Dublin, Ireland

**Abstract:**

 Clear outline on the rationale for this work and a good account of the planned steps for the evaluation within this protocol. Further detail on the methods would strengthen this section - specifically - name the measures designed being used? what qualitative approaches will be used for interview analysis?

**Introduction**

 The distinction between uni-disciplinary and interdisciplinary goal setting and the potential challenges of this is nicely laid out is paragraphs one and two, Paragraph three positions the patient as a shared decision-maker in the goals being set, however, I wonder would it be valuable to a sentence or two that more explicitly considers the patient's unique perspective on goals and priorities for rehabilitation and how this can influence patient engagement and perceptions of QoL?   - Inclusion of this would, I think align well with later points by Doig et al., (2023) about the need to shift towards participation-focused goals.

**Methods**

 Some further development of the qual methods would strengthen this section - themes being explored within the questions and I would suggest inclusion of the semi-structured interview guide as part of the protocol. Development to include how the interview guides were pilot tested, any revisions made. Type of qualitative analysis planned should be considered.

 It is unclear if the two embedded researchers will both complete both qual and quant aspects of data collection or if each are assigned to a single strand. Some development regarding planned steps to minimize bias, ensure a common approach to data collection with the interviews, and reflexivity would be useful within the data collection section.

 "Any changes to the study design will be registered with the protocol." - Is the protocol registered elsewhere? - if so the registration number and site should be added to this paper.

**Recruitment**

 Some further details required on disseminating the study information, voluntary informed consent (for both staff and patients), cooling off periods, steps to reduce perceptions of coercion given the researchers are embedded etc, also given some patients have a cognitive or communication impairment - how will this be navigated in the recruitment and data collection phases? - this point is later somewhat considered in the limitations sections, and I wonder would clearer inclusion criteria assist in clearer consideration of eligibility and decisions around this.

**Overall Comments**

 This protocol addresses an interesting and clinically relevant study question, with the potential to directly inform rehabilitation practice. Overall, the study is well motivated, and the protocol provides a useful foundation for the proposed work.

 Providing greater detail on some aspects of the planned methods and analysis would further strengthen the protocol and enhance clarity for the reader. As I progressed through the full manuscript, I noted that some of my initial queries were subsequently addressed in later sections. This made me wonder whether some resequencing of the manuscript, particularly within the Methods section, might improve the overall flow and ensure that key information is presented closer to where it is first required. Such restructuring may also help to address some of the questions that arose during my reading and make the protocol easier to follow.

**Editing Considerations**

 Suggest editing review so that abbreviations (MDT)  (NRH) (IDT) are used consistently after full terms are first explained.

Is the study design appropriate for the research question?

Yes

Is the rationale for, and objectives of, the study clearly described?

Yes

Are sufficient details of the methods provided to allow replication by others?

Partly

Are the datasets clearly presented in a useable and accessible format?

Partly

Reviewer Expertise:

My background and research experience are in enduring health conditions, service design, and rehabilitation research, including the design and evaluation of studies examining person-centred rehabilitation outcomes and clinical practice.

I confirm that I have read this submission and believe that I have an appropriate level of expertise to confirm that it is of an acceptable scientific standard, however I have significant reservations, as outlined above.

# Response

**Published:** 2026-08-18

**Authors:** Áine Carroll

**Affiliations:** University College Dublin, Ireland

**Response to Reviewer**

 We thank the reviewer for the thoughtful and constructive review and for recognising the clinical relevance of the study, the clarity of its rationale and the appropriateness of the study design. We particularly appreciated the observation that some methodological information became apparent only later in the manuscript. In response, we have strengthened methodological signposting, provided additional detail on recruitment and consent, clarified the qualitative interview domains and the role of the embedded researchers, made reflexivity more explicit, and clarified the abstract. We have also reviewed the manuscript for consistency in the use of abbreviations.

**Abstract**

**Reviewer comment:**

 The abstract provides a clear rationale and overview, but further methodological detail would strengthen it, particularly the names of the measures used and the qualitative analytic approach.

**Response:**

 Thank you. We have revised the Methods section of the abstract to name the four quantitative measures used in the study: the Client-Centredness of Goal Setting scale (C-COGS), CollaboRATE, the Interprofessional Socialization and Valuing Scale (ISVS-9B), and the Normalization Measure Development questionnaire (NoMAD). We have also made explicit that qualitative interview data will be analysed using reflexive thematic analysis.

 The revised abstract now states:

*Self-report measures include the Client-Centredness of Goal Setting scale (C-COGS), CollaboRATE, the Interprofessional Socialization and Valuing Scale (ISVS-9B), and the Normalization Measure Development questionnaire (NoMAD), capturing person-centredness of goals, shared decision-making, interprofessional socialisation and process normalisation. Quantitative data will be analysed using descriptive and, where appropriate, inferential statistics; qualitative interview data will be analysed using reflexive thematic analysis.*

 The original abstract already identified the constructs and reflexive thematic analysis; the revision makes the instruments themselves explicit.

**Introduction**

**Reviewer comment:**

 The reviewer suggested making the patient's unique perspective on rehabilitation goals and priorities more explicit, including how this may influence engagement and participation.

**Response:**

 Thank you. We agree that the particular contribution of the patient's own priorities could be made more explicit. We have therefore added a sentence to the discussion of person-centred goal setting emphasising that meaningful rehabilitation goals should be oriented towards participation and outcomes that matter to the individual rather than being determined solely by professional priorities.

 The following sentence has been added:

*In rehabilitation, this perspective is particularly important because goals that reflect what the person considers meaningful in their own life can orient rehabilitation towards participation and outcomes that matter to them, rather than towards professionally determined impairments or activities alone.*

 We have retained the focus on meaningful goals, participation and engagement rather than introducing quality of life as a separate construct, as quality of life is not an outcome being evaluated in this protocol. The existing Introduction already links person-centred goals with motivation, engagement and goal attainment.

**Methods: qualitative methods and interview guides**

**Reviewer comment:**

 The reviewer requested greater detail on the qualitative methods, including the topics explored in the interviews, development and testing of the interview guides, and the planned qualitative analysis. Inclusion of the semi-structured interview guides was also suggested.

**Response:**

 Thank you. We have expanded the qualitative data collection section to describe more explicitly the areas explored in both patient and staff interviews.

 For patients, the revised text clarifies that open-ended questions explore goal setting, participation in decision-making, relationships with the rehabilitation team, information and communication, and perceived facilitators and challenges across the rehabilitation admission.

 For staff, the revised text clarifies that interviews address interdisciplinary teamwork and interprofessional collaboration, person-centred care, shared decision-making, experiences of the new goal-setting process, and perceived barriers and enablers to implementation and normalisation.

 We have also clarified that the interview guides were developed by the researchers and reviewed and refined through the co-design process, including specific review by former patients and staff representatives. They were not subjected to a separate formal pilot test, and we have not retrospectively described this process as pilot testing.

 The patient and staff semi-structured interview guides have also been included as supplementary material.

 The existing manuscript already specifies the constructionist orientation, reflexive thematic analysis and Braun and Clarke's iterative analytic process; we have strengthened signposting to make this information easier to locate.

**Methods: embedded researchers, consistency and reflexivity**

**Reviewer comment:**

 The reviewer asked for clarification regarding the roles of the two embedded researchers in qualitative and quantitative data collection, and for further detail on consistency of interviewing, potential bias and reflexivity.

**Response:**

 Thank you. We have clarified the role of the embedded research team and added explicit consideration of reflexivity.

 The revised manuscript states that the embedded researchers contribute to data collection across the study as required. Common study procedures and semi-structured topic guides support consistency in data collection while retaining the flexibility appropriate to qualitative interviewing.

 We have also clarified that embedded researchers conducting patient interviews are not members of participants’ treating teams. Given their embedded position within the implementation team, we have added explicit consideration of how prior relationships, organisational knowledge and researcher assumptions may shape data generation and interpretation.

 We have deliberately framed this issue in terms of reflexivity rather than attempting to suggest that researcher subjectivity can be eliminated, consistent with the constructionist orientation and reflexive thematic analysis adopted in the protocol.

 The later conduct of the patient study confirms the distinction between researchers' embedded implementation role and the participants' treating teams, although the protocol itself retains the personnel and roles prospectively specified at the outset.

**Methods: protocol registration**

**Reviewer comment:**

 The reviewer queried the statement that changes to the study design would be “registered with the protocol” and asked whether the protocol was registered elsewhere.

**Response:**

 Thank you for identifying this ambiguity. The wording did not refer to a separate trial or protocol registration.

 We have replaced the sentence with:

*Any substantive changes to the study design will be documented in subsequent versions of this protocol and reported transparently in publications arising from the study.*

 This more accurately reflects the intended meaning and removes any implication of a separate registration. The original wording appeared in the Study Design section.

**Recruitment and informed consent**

**Reviewer comment:**

 The reviewer requested additional detail regarding provision of study information, voluntary informed consent, time to consider participation, potential perceptions of coercion associated with the embedded researcher role, and recruitment of patients with cognitive or communication impairments.

**Response:**

 Thank you. We agree that this aspect of the protocol would benefit from greater procedural detail. We have therefore added a dedicated Recruitment and Consent subsection.

 The revised section describes provision of written study information, subsequent explanation of the study by a researcher, the opportunity to ask questions and consider participation before consent is obtained, and the voluntary nature of participation. It also makes clear that participation or non-participation will not affect clinical care or staff employment/professional relationships.

 A prescribed formal cooling-off period was not a feature of the approved protocol and we have therefore not retrospectively introduced one. Instead, we have clarified that potential participants have an opportunity to consider the information and ask questions before deciding whether to participate.

 The consent procedures described are consistent with the study as subsequently conducted, in which potential patient participants received study information before researcher contact and were allowed time for consideration prior to consent.

**Recruitment of patients with cognitive or communication impairment**

**Reviewer comment:**

 The reviewer asked for clearer consideration of how cognitive and communication impairments would be managed during recruitment and data collection.

**Response:**

 Thank you. The protocol already included provisions for supported participation, but we agree that these should be more prominent within the recruitment and consent process.

 We have therefore strengthened the cross-referencing between the Eligibility Criteria and Recruitment and Consent sections. The manuscript clarifies that the presence of a cognitive or communication impairment does not in itself result in exclusion. Existing hospital-approved communication supports, including communication passports, augmentative and alternative communication aids and family/carer-supported participation, may be used where appropriate. Exclusion occurs only where available supports are insufficient to enable valid consent and meaningful participation and to elicit the patient's own view.

**Organisation and sequencing of the Methods**

**Reviewer comment:**

 The reviewer observed that some questions arising during the initial reading were answered later in the manuscript and suggested that some resequencing might improve flow.

**Response:**

 We agree and thank the reviewer for this helpful observation. Rather than substantially restructuring the Methods section, which broadly follows the sequence of the study, we have improved signposting and placed recruitment and consent information in a dedicated subsection adjacent to the eligibility criteria.

 We have also added clearer cross-referencing from Study Design to the subsequent Qualitative Data and Data Analysis sections so that readers are directed to the relevant methodological detail when each component is first introduced.

 This addresses the reviewer’s concern while avoiding unnecessary duplication of methodological information already provided later in the manuscript. The reviewer similarly noted that several initial queries were answered as the manuscript progressed.

**Abbreviations**

**Reviewer comment:**

 The reviewer recommended consistent use of abbreviations such as MDT, IDT and NRH after the terms have first been defined.

**Response:**

 Thank you. We have reviewed the manuscript and standardised the use of abbreviations throughout following their first definition.

**Availability of datasets**

**Reviewer assessment:**

 Datasets were rated as only partly presented in a usable and accessible format.

**Response:**

 We note that this article reports a study protocol rather than study findings. Consequently, no study dataset is associated with the protocol article. The Data Availability statement therefore remains:

 No data are associated with this article.

 The protocol currently states this explicitly.

**Overall comments**

**Reviewer comment:**

 The reviewer considered the study clinically relevant and well-motivated and suggested that additional methodological detail would strengthen the protocol.

**Response:**

 We thank the reviewer for these constructive comments. We believe that the revisions have improved the clarity and transparency of the protocol while preserving its prospective nature. The protocol was developed and published before data collection commenced; accordingly, the revisions clarify the originally planned study rather than retrospectively altering the protocol to reflect subsequent implementation changes. The later study manuscript explicitly records that the protocol preceded data collection.

 We thank the reviewer again for the careful reading of the manuscript and for the suggestions, which have improved both the methodological clarity and navigability of the protocol.

# Reviewer report: Reviewer response for version 2

**DOI:** 10.21956/hrbopenres.15995.r56724

**Published:** 2026-07-29

**Authors:** Yuho Okita

**Affiliations:** 1 Swinburne University of Technology, Hawthorn, Australia

All the questions raised earlier have now been addressed and answered.

Is the study design appropriate for the research question?

Partly

Is the rationale for, and objectives of, the study clearly described?

Partly

Are sufficient details of the methods provided to allow replication by others?

Partly

Are the datasets clearly presented in a useable and accessible format?

Partly

Reviewer Expertise:

NA

I confirm that I have read this submission and believe that I have an appropriate level of expertise to confirm that it is of an acceptable scientific standard.

# Reviewer report: Reviewer response for version 1

**DOI:** 10.21956/hrbopenres.14985.r43546

**Published:** 2024-12-27

**Authors:** Yuho Okita

**Affiliations:** 1 Swinburne University of Technology, Hawthorn, Australia

I have several questions and points of clarification regarding your research:

 Research Questions: While I can see that research questions 1–3 can be addressed through the qualitative aspects of your mixed-methods approach, what specific research question are you trying to answer with the quantitative aspects?

 Goal Setting Process: In the abstract, you mentioned: "The goal-setting process was collaboratively designed based on iterative rounds of patient and staff feedback." Does this mean the goal-setting process has already been developed in prior work, and this study aims to refine that process through additional research?

 Qualitative and Quantitative Rationale: You highlighted the rationale for using qualitative methods by stating: "By capturing staff and patient experiences using multiple and mixed methods, we can gain insight into the phenomenon of shared decision-making, IDT goal setting from different perspectives, as well as barriers to implementation in this context." However, why is there also a need for quantitative methods? Where is the specific rationale for including this aspect?

 Recruitment and Eligibility: Who will be responsible for recruiting participants and determining their eligibility?

 Previous Research: You wrote: "The new goal process was developed in response to feedback from and in collaboration with NRH staff and patient representatives." Was this process developed in your previous research?

 Clarify Researcher Roles: For the analysis section, you mentioned that the lead researcher will conduct the analysis. This needs to be clearly stated in other sections, such as recruitment and participant eligibility determination, to ensure consistency and clarity.

 Definition of "Staff": What does “staff” refer to in this context? Are you including rehabilitation practitioners, carers, doctors, nurses, or other groups?

 Overall Aim of the Research: I did not fully grasp the overall aim of this research. Are you testing the already-developed goal-setting process from a previous study with the intent to refine it? Or are you exploring participants' experiences of current shared decision-making practices to identify areas for improvement? Or is it a combination of both?

Is the study design appropriate for the research question?

Partly

Is the rationale for, and objectives of, the study clearly described?

Partly

Are sufficient details of the methods provided to allow replication by others?

Partly

Are the datasets clearly presented in a useable and accessible format?

Partly

Reviewer Expertise:

NA

I confirm that I have read this submission and believe that I have an appropriate level of expertise to confirm that it is of an acceptable scientific standard, however I have significant reservations, as outlined above.

# Response

**Published:** 2026-07-06

**Authors:** Áine Carroll

**Affiliations:** University College Dublin, Ireland

**Comment 1: Research questions and the quantitative aspects.** *“What specific research question are you trying to answer with the quantitative aspects?”*

**Response:** This was underspecified in the original submission. We have added a sentence following Research Question 2 clarifying that the quantitative measures (ISVS-9B, NoMAD) provide standardised, repeated-measures data on staff interprofessional socialisation and process normalisation, which complements the qualitative interviews by allowing us to track group-level trends over time and to triangulate these trends against individual staff narratives. This addition appears in the “Research questions, aims and objectives” section.

**Comment 2: Was the goal-setting process developed in prior work?**

**Response:** Yes, and we agree this was not made explicit. We have added a clarifying statement at the start of the “Intervention” section noting that the new goal-setting process was developed prior to, and separately from, the current evaluation study, through a quality improvement initiative led by the NRH’s IDT Quality Care Team in collaboration with staff and patient representatives. The present protocol concerns the evaluation of that process, not its development.

**Comment 3: Rationale for quantitative methods alongside qualitative.**

**Response: **We have expanded the rationale in the “Study design” section to explain that the quantitative and qualitative strands serve complementary purposes: qualitative interviews provide depth of understanding of individual experience, while quantitative measures allow us to track standardised change over time across the full staff group and to assess feasibility for a future hospital-wide impact evaluation. We have also added a description, in the Data analysis section, of how the two strands will be integrated at the interpretation stage.

**Comment 4: Recruitment and eligibility responsibility.**

**Response: **We have added a sentence to the “Eligibility criteria” section clarifying that recruitment and eligibility screening will be carried out jointly by the embedded researchers (LC, ZT) and clinical staff (e.g. the named keyworker or team coordinator) on each participating unit.

**Comment 5: Was the goal process developed in the authors’ previous research?**

**Response: **As above (Comment 2), we have clarified that the process was developed through a separate, prior quality improvement initiative at NRH, not through prior academic research by this author team. We have added detail on who was involved in that development (staff across disciplines and patient representatives, coordinated by the IDT Quality Care Team).

**Comment 6: Consistency in describing researcher roles.**

**Response:** We have added a sentence to the “Study design” section stating that the embedded researchers (LC, ZT) hold primary responsibility across recruitment, data collection and analysis, working alongside clinical staff for participant identification, so that this is stated consistently rather than only in the analysis section.

**Comment 7: Definition of “staff”.**

**Response:** We have added a definition to the “Eligibility criteria” section: for this study, “staff” refers to clinical and allied health professionals directly involved in patient care and the goal setting process on participating units (medical, nursing, health care assistant (HCA) and health and social care professional (HSCP) staff) and does not include non-clinical or administrative staff.

**Comment 8: Overall aim of the research.**

**Response:** We recognise the original text did not make sufficiently clear that this is a combination of both evaluating the already co-designed process and exploring current staff/patient experience of shared decision-making practice as it is now being used. We have revised the closing paragraph of the introduction and the opening of the “Research questions, aims and objectives” section to state explicitly that the study aims (a) to evaluate staff and patient experience of the process as implemented, and (b) to use this evaluation to identify barriers and enablers that can inform iterative refinement and future hospital-wide rollout.

# Reviewer report: Reviewer response for version 1

**DOI:** 10.21956/hrbopenres.14985.r43547

**Published:** 2024-12-26

**Authors:** Lesley Scobbie

**Affiliations:** 1 Glasgow Caledonian university, Glasgow, UK

**Pilot of a person-centred, interdisciplinary approach to goal setting in Ireland’s National Rehabilitation Hospital: a study protocol**

 The outlined study seeks to use a mixed methods approach to evaluate the introduction of a tailored, shared decision-making, interdisciplinary goal setting process with a mixed diagnostic group of patients in an inpatient rehabilitation setting in Ireland, including identifying barriers and enablers to its implementation.

***General points:***

 > Avoid use of too many abbreviations as it gets confusing for the reader.

 > There are many different concepts being evaluation in this study – interdisciplinary teamwork, shared decision making, person-centred care, goal setting and implementation. Each of these concepts should be defined (some are) and how they will be evaluated unpacked on an individual basis.

 > No details have been provided re. staff training to use the new goal setting approach - this is a significant omission. 

***Abstract*:**

 Would have been helpful to write IDT in full i.e. interdisciplinary team, as it is not clear what it stands for within the abstract.

***Introduction:***

 Para2: Write MDT in full at first mention.

 Clear definition of Multi-Disciplinary Team would be helpful in the introduction.

***Methods:***

 Re. Eligibility criteria – *“Patients will be excluded if they have severe cognitive or communication impairments resulting in the inability to participate in the interview”*

 To optimise inclusivity, are there any supports that can be put in place to support people with cognitive and / or communication difficulties to participate in this research?

 Re. Sampling - What kind of sampling method will be used to inform patient and staff recruitment?

 Re. Intervention – Are there any details about how the new goal process was developed? How many staff and patients were involved in its development and who were they?

 As noted in the introduction, goal setting approaches have been developed and reported e.g., Scobbie et al 2020; Van De Weyer 2010; Doig et al 2023.  It would be helpful if the authors could justify development of their own approach rather than build on one of those already developed.

 Figure 1 is helpful but does not fully describe the new goal setting approach.

 For example,

- What is the information booklet that is given out pre admission. Will there be a different one for each specialty?
- How will an individual’s readiness/ desires to lead own goals? Would it not be appropriate at times for a staff member to lead if the patient is struggling/ overwhelmed/ lacking confidence?
- What are the recognised tools and accessible support proposed for use at a meet the team session?
- Should the patient not be involved in deciding what is required to support progress towards their goals (given it is a person centred approach)?
- Team and family feedback on goal outcomes; should the patient be involved oi this too?
- Would TiDIER have been a better way to describe the intervention? https://www.equator-network.org/wp-content/uploads/2014/03/TIDieR-Checklist-PDF.pdf?

Re. Data Collection

 “(C-Cogs and CollaboRATE) will be administered shortly after the initial goal setting meeting”

 It is unclear from Fig 1. what this meeting is – meet the team/ goal setting conference?

 Will there be support available if for any reason patients find these measure difficult to complete?

 Will and if so, how will the qualitative and quantitative data be combined/ compared/ joined?

 Re. Public and Patient Involvement – it is notable only two patients were involved (no info given re their background/ level of involvement) and no carers. Given this goal setting process may be used with a variety of patients with different ages/ diagnosis - this seems to be a small number.

Is the study design appropriate for the research question?

Partly

Is the rationale for, and objectives of, the study clearly described?

Yes

Are sufficient details of the methods provided to allow replication by others?

No

Are the datasets clearly presented in a useable and accessible format?

Not applicable

Reviewer Expertise:

goal setting, stroke rehabilitation, implementation

I confirm that I have read this submission and believe that I have an appropriate level of expertise to state that I do not consider it to be of an acceptable scientific standard, for reasons outlined above.

# Response

**Published:** 2026-07-06

**Authors:** Áine Carroll

**Affiliations:** University College Dublin, Ireland

**General points**

***Abbreviations:*** We have reviewed the manuscript throughout and expanded abbreviations at first use that had not previously been defined (notably HCA and HSCP), while retaining abbreviations that are standard and necessary for readability (e.g. NRH, IDT, EPR) once defined.

***Multiple concepts requiring individual definition:*** We agree that interdisciplinary teamwork, shared decision-making, person-centred care, goal setting and implementation/normalisation are distinct constructs. We have added or sharpened definitions for each on first use in the Introduction, and have clarified, where each construct is operationalised, which specific measure or interview topic captures it (see Methods).

***Staff training omission:*** This was a significant gap, and we thank the reviewer for identifying it. We have added a new subsection describing staff training delivered prior to implementation, covering the documentation, the underlying principles of person-centred and shared decision-making practice, and use of the pre-admission information booklet, delivered via team-based workshops facilitated by the IDT Quality Care Team.

**Abstract**

 We have spelled out “interdisciplinary team (IDT)” in full at first mention in the Abstract.

**Introduction**

 We have written “multidisciplinary team (MDT)” in full at first mention (paragraph 1) and added a short comparative definition distinguishing MDT and IDT working, so that the abbreviation used later in paragraph 2 is already defined.

**Methods**

*Eligibility criteria / inclusivity for patients with cognitive or communication impairments: *We have added a statement describing supports that will be offered to patients with mild-to-moderate cognitive or communication difficulties who do not meet exclusion criteria, including additional time, Easy-Read materials, communication aids, and involvement of a family member or carer to support participation.

*Sampling method: *We have added a short statement specifying that a convenience sampling approach will be used for both staff and patients, given the relatively small potential participant pool across the three pilot units.

*Development of the new goal process / justification relative to existing frameworks: *We have added a paragraph to the “Intervention” section acknowledging the existing frameworks the reviewer notes (G-AP, the keyworker-led model, the role-based IDT approach) and explaining that, consistent with evidence that complex interventions are best tailored to local context (Craig et al., 2013, already cited), the NRH team elected to co-design a context-specific process informed by, but not a direct replication of, these frameworks, reflecting NRH’s mixed case-mix and its concurrent transition to an electronic patient record.

*Figure 1 clarifications:* We have added explanatory text addressing each point raised: - The pre-admission information booklet: a core version is used hospital-wide, with programme-specific inserts for each speciality (stroke, ABI, SCSC, POLAR, paediatric). - Readiness to lead own goals: we clarify that a staff member may take a more directive lead where a patient’s readiness or capacity is limited, with the explicit aim of increasing patient control as capacity develops. - Recognised tools and accessible supports: we now specify examples (Easy-Read materials, communication charts/boards, interpreter or speech and language therapy input) selected according to individual need. - Patient involvement in support planning: we clarify that patients and families are invited to contribute to decisions about the tasks/activities/timetable required to support progress towards their goals. - Family feedback on goal outcomes: we clarify that patients and families are involved together in this feedback at the pre-discharge meeting.

*TIDieR checklist*: We agree this is a useful reference point for intervention reporting and have added a note in the Intervention section that the description follows relevant elements of the TIDieR checklist (Hoffmann et al., 2014) to support replicability, while retaining our narrative + figure format so as not to substantially restructure the protocol.

*Data collection*, *meeting terminology*. We have clarified that the “initial goal setting meeting” referred to in the measures section corresponds to the goal, action and care planning (goal setting conference) meeting shown in Figure 1.

*Support for patients completing self-report measures: *We have added a statement that the embedded researcher will offer support (reading items aloud, additional time, or recording responses on the patient’s behalf) without altering item content or interpretation, for patients who require it.

*Combining/joining qualitative and quantitative data*: We have added a statement to the Data analysis section describing that the two strands will be integrated at the interpretation stage using a mixed-methods triangulation approach, with joint displays used where relevant to explore convergence or divergence between self-report measures and interview themes.

*Public and Patient Involvement:* We have expanded this section to give brief, anonymised background on the two patient co-researchers’ prior admissions and have added an explicit acknowledgement that PPI in this phase was limited to two individuals with lived experience and did not include family carers, with a commitment to broadening patient and carer involvement in the subsequent hospital-wide implementation study.

 We thank the reviewer for their thorough and helpful engagement with this protocol, which we believe has considerably improved the clarity and rigour of the study design.
